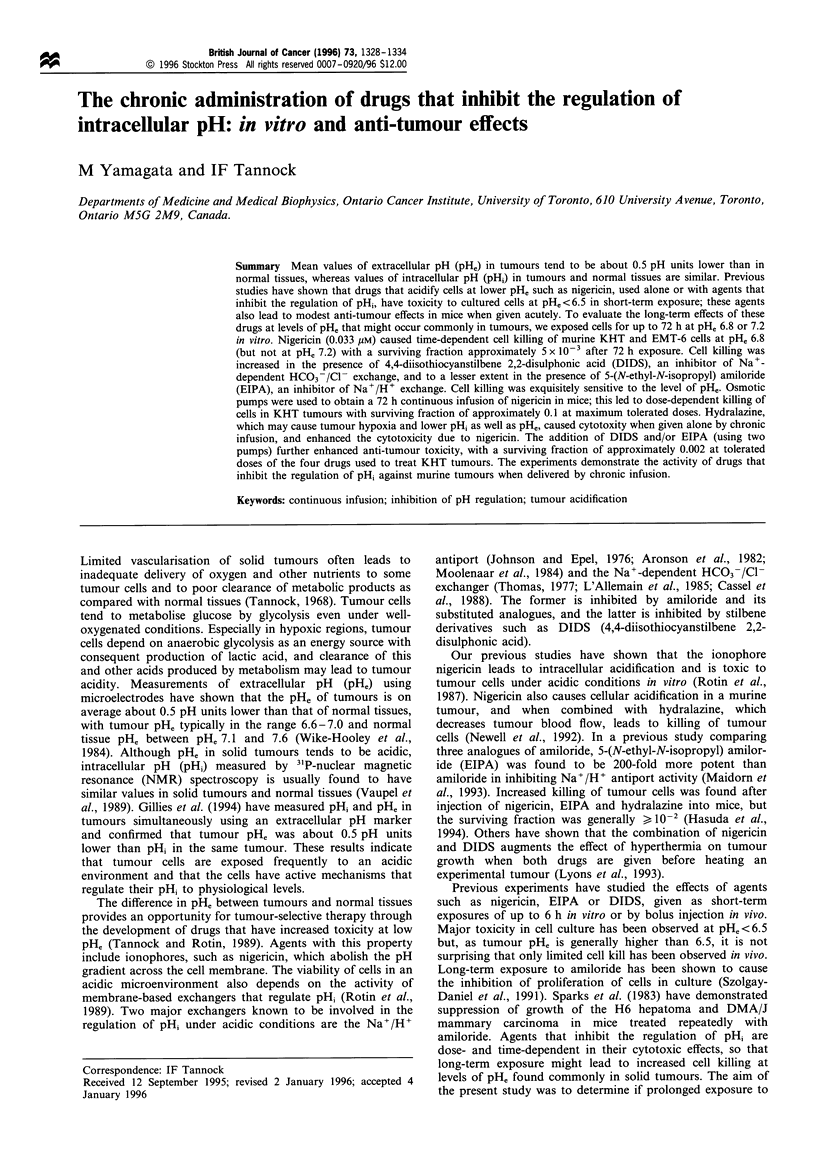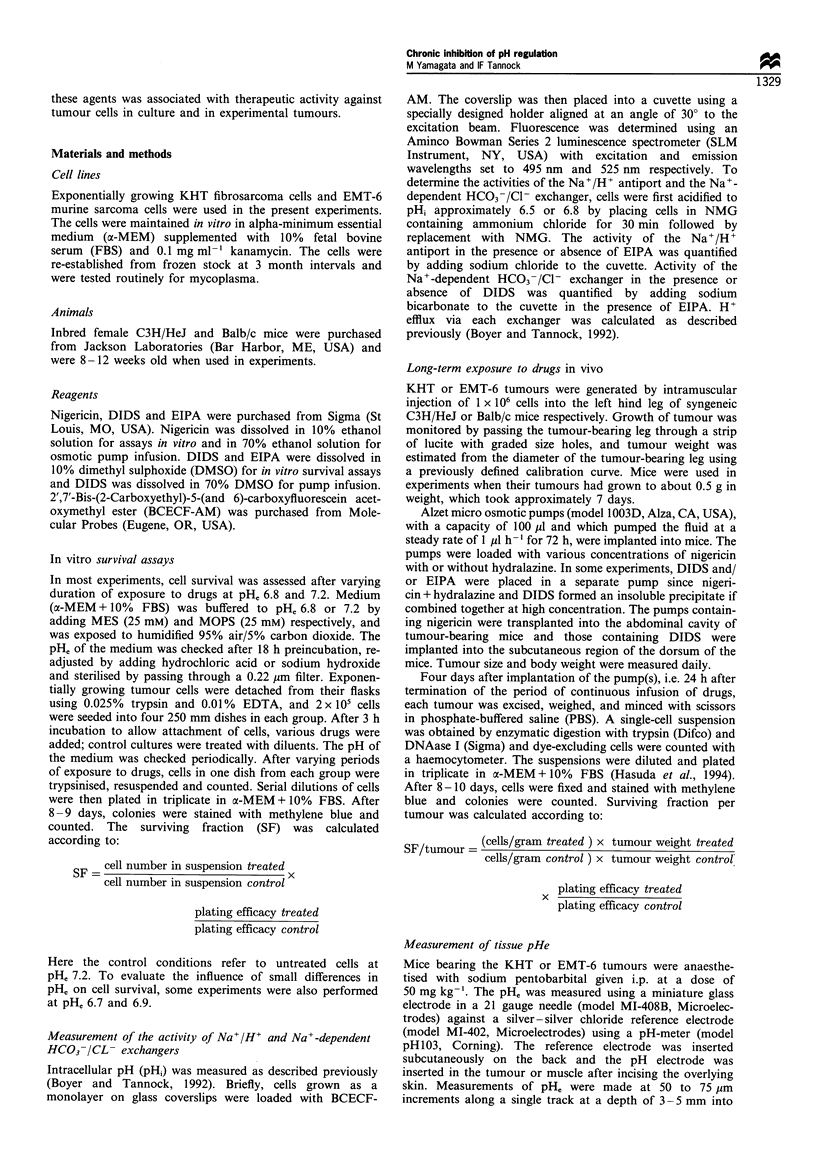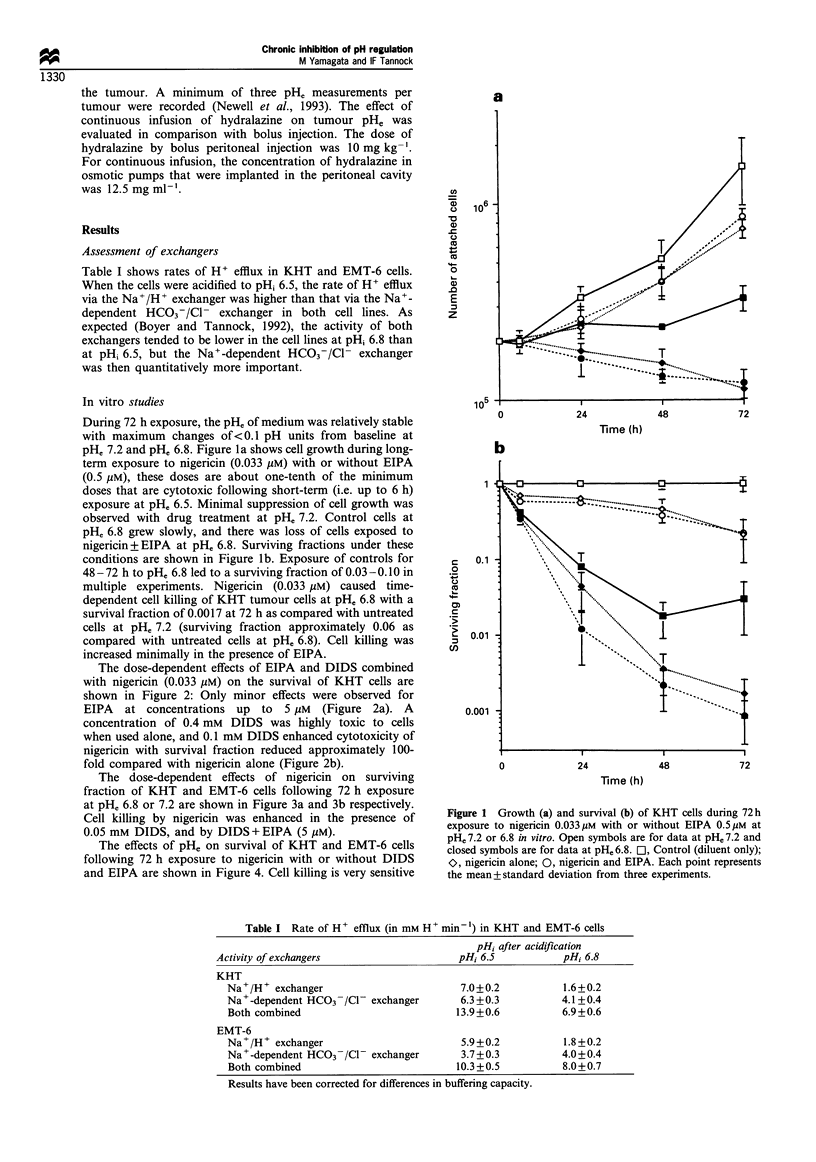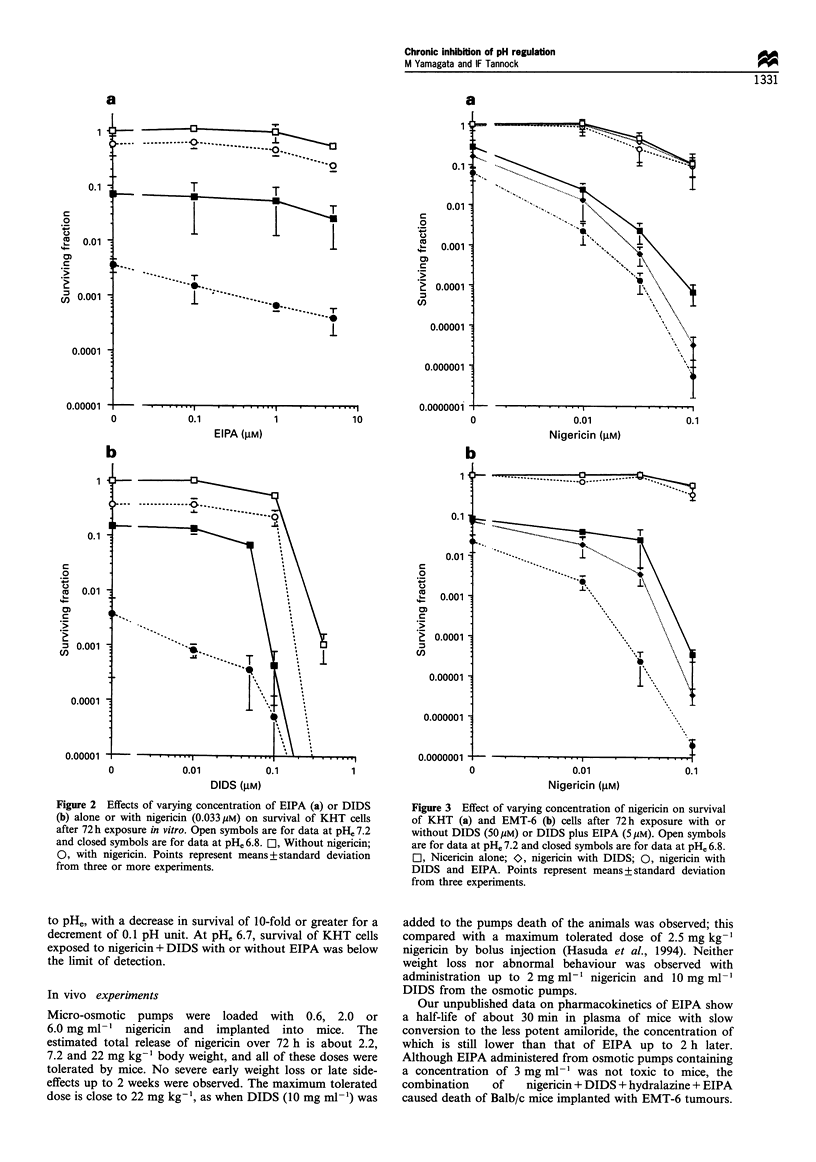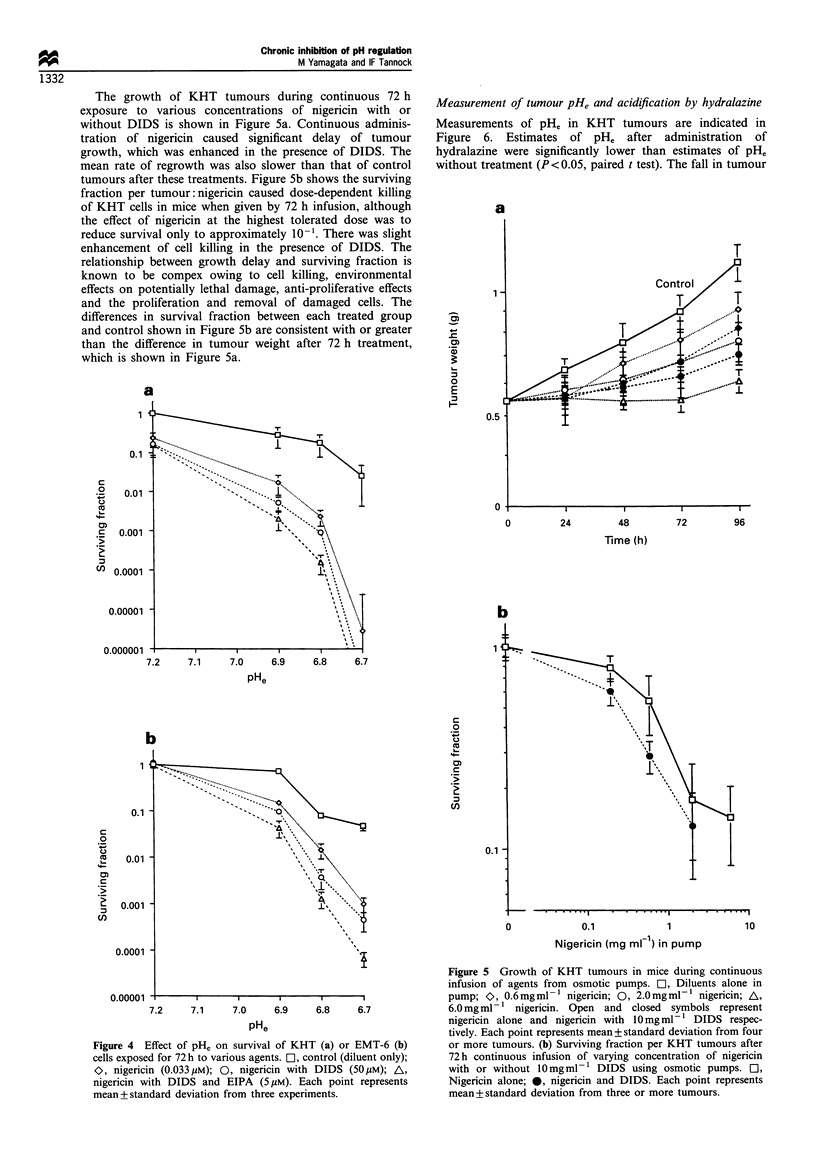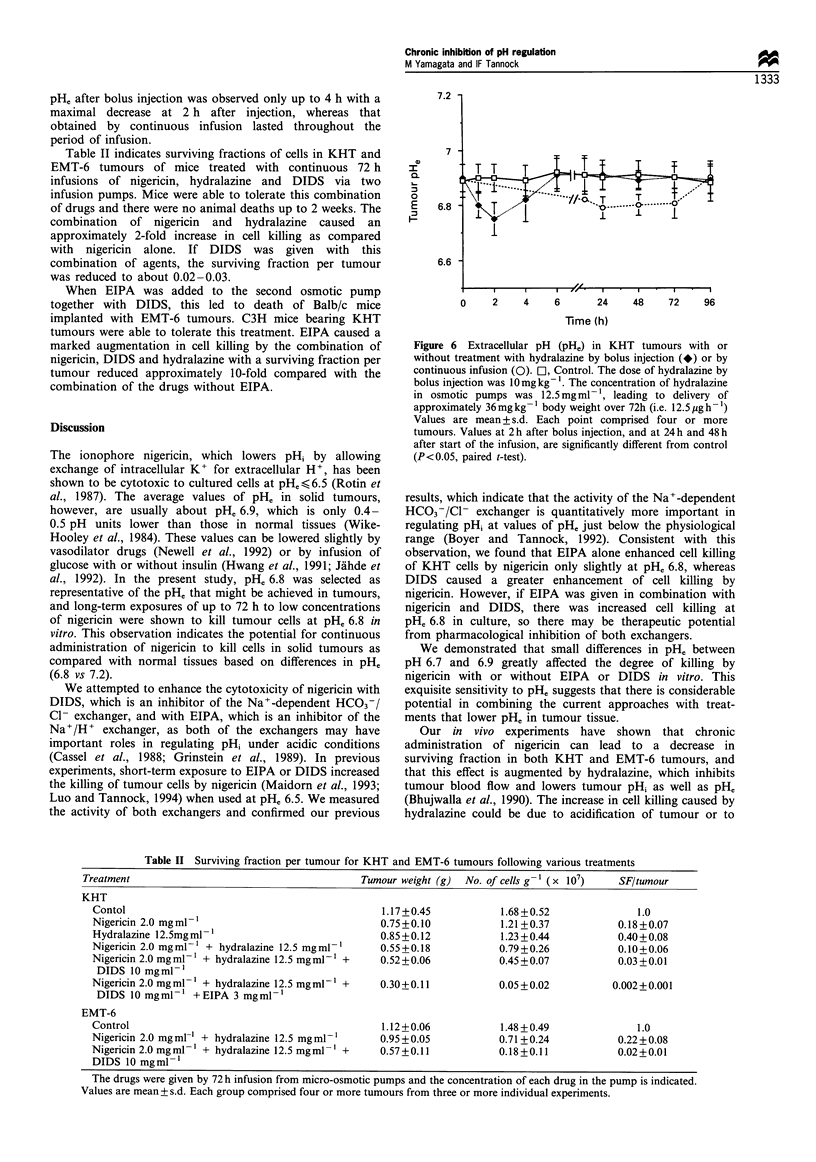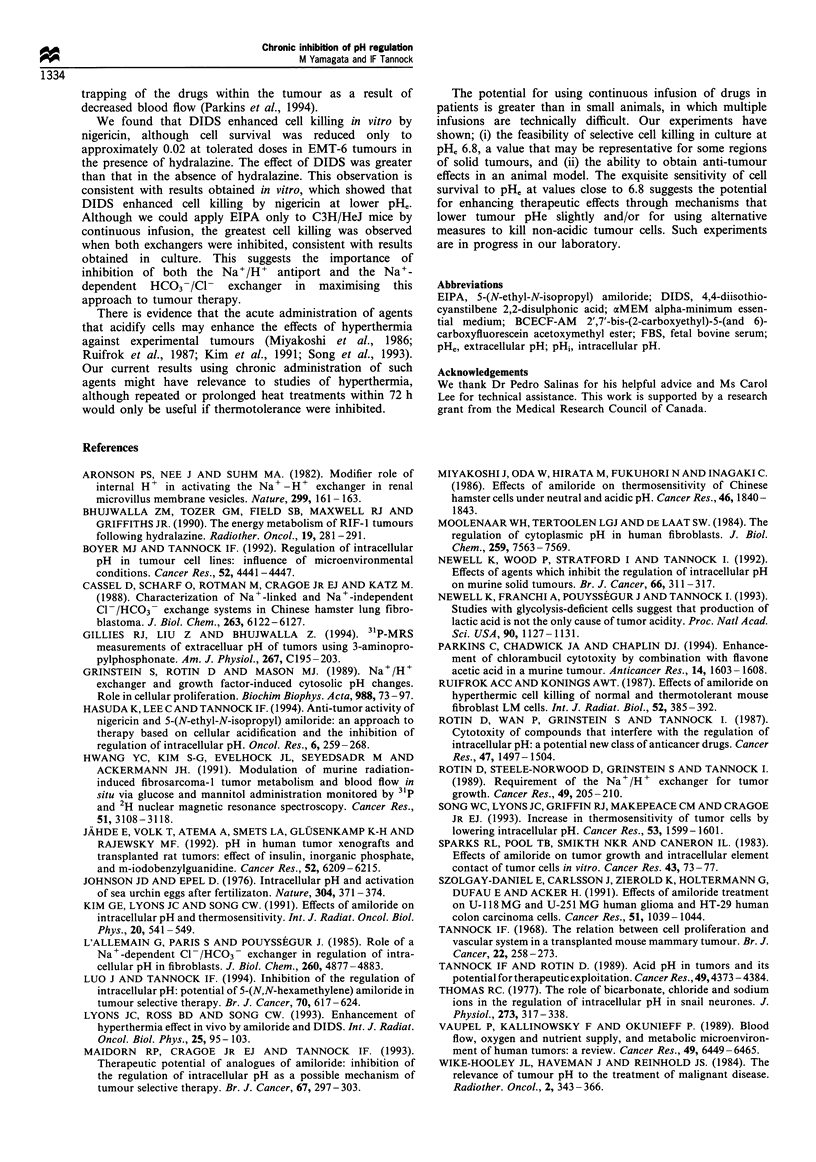# The chronic administration of drugs that inhibit the regulation of intracellular pH: in vitro and anti-tumour effects.

**DOI:** 10.1038/bjc.1996.254

**Published:** 1996-06

**Authors:** M. Yamagata, I. F. Tannock

**Affiliations:** Department of Medicine, Ontario Cancer Institute, University of Toronto, Canada.

## Abstract

Mean values of extracellular pH (pHe) in tumours tend to be about 0.5 pH units lower than in normal tissues, whereas values of intracellular pH (pHi) in tumours and normal tissues are similar. Previous studies have shown that drugs that acidify cells at lower pHe such as nigericin, used alone or with agents that inhibit the regulation of pHi, have toxicity to cultured cells at pHe < 6.5 in short-term exposure; these agents also lead to modest anti-tumour effects in mice when given acutely. To evaluate the long-term effects of these drugs at levels of pHe that might occur commonly in tumours, we exposed cells for up to 72h at pHe 6.8 or 7.2 in vitro. Nigericin (0.033 microM) caused time-dependent cell killing of murine KHT and EMT-6 cells at pHe 6.8 (but not at pHe 7.2) with a surviving fraction approximately 5 x 10(-3) after 72 h exposure. Cell killing was increased in the presence of 4,4-diisothiocyanstilbene 2,2-disulphonic acid (DIDS), an inhibitor of Na+-dependent HCO3-/CI- exchange, and to a lesser extent in the presence of 5-(N-ethyl-N-isopropyl) amiloride (EIPA), an inhibitor of Na+/H+ exchange. Cell killing was exquisitely sensitive to the level of pHe. Osmotic pumps were used to obtain a 72 h continuous infusion of nigericin in mice; this led to dose-dependent killing of cells in KHT tumours with surviving fraction of approximately 0.1 at maximum tolerated doses. Hydralazine, which may cause tumour hypoxia and lower pHi as well as pHe, caused cytotoxity when given alone by chronic infusion, and enhanced the cytotoxicity due to nigericin. The addition of DIDS and/or EIPA (using two pumps) further enhanced anti-tumour toxicity, with a surviving fraction of approximately 0.002 at tolerated doses of the four drugs used to treat KHT tumours. The experiments demonstrate the activity of drugs that inhibit the regulation of pHi against murine tumours when delivered by chronic infusion.


British Journal of Cancer (1996) 73, 1328-1334
© 1996 Stockton Press All rights reserved 0007-0920/96 $12.00

### The chronic administration of drugs that inhibit the regulation of intracellular pH: *in vitro* and anti-tumour effects

M Yamagata and IF Tannock

*Departments of Medicine and Medical Biophysics, Ontario Cancer Institute, University of Toronto, 610 University Avenue, Toronto, Ontario M5G 2M9, Canada.*

**Summary** Mean values of extracellular pH ($pH_e$) in tumours tend to be about 0.5 pH units lower than in normal tissues, whereas values of intracellular pH ($pH_i$) in tumours and normal tissues are similar. Previous studies have shown that drugs that acidify cells at lower $pH_e$ such as nigericin, used alone or with agents that inhibit the regulation of $pH_i$, have toxicity to cultured cells at $pH_e < 6.5$ in short-term exposure; these agents also lead to modest anti-tumour effects in mice when given acutely. To evaluate the long-term effects of these drugs at levels of $pH_e$ that might occur commonly in tumours, we exposed cells for up to 72 h at $pH_e$ 6.8 or 7.2 *in vitro*. Nigericin (0.033 $\mu$M) caused time-dependent cell killing of murine KHT and EMT-6 cells at $pH_e$ 6.8 (but not at $pH_e$ 7.2) with a surviving fraction approximately $5 \times 10^{-3}$ after 72 h exposure. Cell killing was increased in the presence of 4,4-diisothiocyanstilbene 2,2-disulphonic acid (DIDS), an inhibitor of $Na^+$-dependent $HCO_3^-/Cl^-$ exchange, and to a lesser extent in the presence of 5-(*N*-ethyl-*N*-isopropyl) amiloride (EIPA), an inhibitor of $Na^+/H^+$ exchange. Cell killing was exquisitely sensitive to the level of $pH_e$. Osmotic pumps were used to obtain a 72 h continuous infusion of nigericin in mice; this led to dose-dependent killing of cells in KHT tumours with surviving fraction of approximately 0.1 at maximum tolerated doses. Hydralazine, which may cause tumour hypoxia and lower $pH_i$ as well as $pH_e$, caused cytotoxity when given alone by chronic infusion, and enhanced the cytotoxicity due to nigericin. The addition of DIDS and/or EIPA (using two pumps) further enhanced anti-tumour toxicity, with a surviving fraction of approximately 0.002 at tolerated doses of the four drugs used to treat KHT tumours. The experiments demonstrate the activity of drugs that inhibit the regulation of $pH_i$ against murine tumours when delivered by chronic infusion.

**Keywords:** continuous infusion; inhibition of pH regulation; tumour acidification

Limited vascularisation of solid tumours often leads to inadequate delivery of oxygen and other nutrients to some tumour cells and to poor clearance of metabolic products as compared with normal tissues (Tannock, 1968). Tumour cells tend to metabolise glucose by glycolysis even under well-oxygenated conditions. Especially in hypoxic regions, tumour cells depend on anaerobic glycolysis as an energy source with consequent production of lactic acid, and clearance of this and other acids produced by metabolism may lead to tumour acidity. Measurements of extracellular pH ($pH_e$) using microelectrodes have shown that the $pH_e$ of tumours is on average about 0.5 pH units lower than that of normal tissues, with tumour $pH_e$ typically in the range 6.6–7.0 and normal tissue $pH_e$ between $pH_e$ 7.1 and 7.6 (Wike-Hooley *et al.*, 1984). Although $pH_e$ in solid tumours tends to be acidic, intracellular pH ($pH_i$) measured by $^{31}P$-nuclear magnetic resonance (NMR) spectroscopy is usually found to have similar values in solid tumours and normal tissues (Vaupel *et al.*, 1989). Gillies *et al.* (1994) have measured $pH_i$ and $pH_e$ in tumours simultaneously using an extracellular pH marker and confirmed that tumour $pH_e$ was about 0.5 pH units lower than $pH_i$ in the same tumour. These results indicate that tumour cells are exposed frequently to an acidic environment and that the cells have active mechanisms that regulate their $pH_i$ to physiological levels.

The difference in $pH_e$ between tumours and normal tissues provides an opportunity for tumour-selective therapy through the development of drugs that have increased toxicity at low $pH_e$ (Tannock and Rotin, 1989). Agents with this property include ionophores, such as nigericin, which abolish the pH gradient across the cell membrane. The viability of cells in an acidic microenvironment also depends on the activity of membrane-based exchangers that regulate $pH_i$ (Rotin *et al.*, 1989). Two major exchangers known to be involved in the regulation of $pH_i$ under acidic conditions are the $Na^+/H^+$ antiport (Johnson and Epel, 1976; Aronson *et al.*, 1982; Moolenaar *et al.*, 1984) and the $Na^+$-dependent $HCO_3^-/Cl^-$ exchanger (Thomas, 1977; L'Allemain *et al.*, 1985; Cassel *et al.*, 1988). The former is inhibited by amiloride and its substituted analogues, and the latter is inhibited by stilbene derivatives such as DIDS (4,4-diisothiocyanstilbene 2,2-disulphonic acid).

Our previous studies have shown that the ionophore nigericin leads to intracellular acidification and is toxic to tumour cells under acidic conditions *in vitro* (Rotin *et al.*, 1987). Nigericin also causes cellular acidification in a murine tumour, and when combined with hydralazine, which decreases tumour blood flow, leads to killing of tumour cells (Newell *et al.*, 1992). In a previous study comparing three analogues of amiloride, 5-(*N*-ethyl-*N*-isopropyl) amiloride (EIPA) was found to be 200-fold more potent than amiloride in inhibiting $Na^+/H^+$ antiport activity (Maidorn *et al.*, 1993). Increased killing of tumour cells was found after injection of nigericin, EIPA and hydralazine into mice, but the surviving fraction was generally $\geqslant 10^{-2}$ (Hasuda *et al.*, 1994). Others have shown that the combination of nigericin and DIDS augments the effect of hyperthermia on tumour growth when both drugs are given before heating an experimental tumour (Lyons *et al.*, 1993).

Previous experiments have studied the effects of agents such as nigericin, EIPA or DIDS, given as short-term exposures of up to 6 h *in vitro* or by bolus injection *in vivo*. Major toxicity in cell culture has been observed at $pH_e < 6.5$ but, as tumour $pH_e$ is generally higher than 6.5, it is not surprising that only limited cell kill has been observed *in vivo*. Long-term exposure to amiloride has been shown to cause the inhibition of proliferation of cells in culture (Szolgay-Daniel *et al.*, 1991). Sparks *et al.* (1983) have demonstrated suppression of growth of the H6 hepatoma and DMA/J mammary carcinoma in mice treated repeatedly with amiloride. Agents that inhibit the regulation of $pH_i$ are dose- and time-dependent in their cytotoxic effects, so that long-term exposure might lead to increased cell killing at levels of $pH_e$ found commonly in solid tumours. The aim of the present study was to determine if prolonged exposure to

Correspondence: IF Tannock
Received 12 September 1995; revised 2 January 1996; accepted 4 January 1996

these agents was associated with therapeutic activity against tumour cells in culture and in experimental tumours.

### Materials and methods

### *Cell lines*

Exponentially growing KHT fibrosarcoma cells and EMT-6 murine sarcoma cells were used in the present experiments. The cells were maintained *in vitro* in alpha-minimum essential medium (α-MEM) supplemented with 10% fetal bovine serum (FBS) and 0.1 mg ml$^{-1}$ kanamycin. The cells were re-established from frozen stock at 3 month intervals and were tested routinely for mycoplasma.

### *Animals*

Inbred female C3H/HeJ and Balb/c mice were purchased from Jackson Laboratories (Bar Harbor, ME, USA) and were 8–12 weeks old when used in experiments.

### *Reagents*

Nigericin, DIDS and EIPA were purchased from Sigma (St Louis, MO, USA). Nigericin was dissolved in 10% ethanol solution for assays *in vitro* and in 70% ethanol solution for osmotic pump infusion. DIDS and EIPA were dissolved in 10% dimethyl sulphoxide (DMSO) for *in vitro* survival assays and DIDS was dissolved in 70% DMSO for pump infusion. 2′,7′-Bis-(2-Carboxyethyl)-5-(and 6)-carboxyfluorescein acetoxymethyl ester (BCECF-AM) was purchased from Molecular Probes (Eugene, OR, USA).

### In vitro *survival assays*

In most experiments, cell survival was assessed after varying duration of exposure to drugs at $pH_e$ 6.8 and 7.2. Medium (α-MEM + 10% FBS) was buffered to $pH_e$ 6.8 or 7.2 by adding MES (25 mM) and MOPS (25 mM) respectively, and was exposed to humidified 95% air/5% carbon dioxide. The $pH_e$ of the medium was checked after 18 h preincubation, re-adjusted by adding hydrochloric acid or sodium hydroxide and sterilised by passing through a 0.22 μm filter. Exponentially growing tumour cells were detached from their flasks using 0.025% trypsin and 0.01% EDTA, and $2 \times 10^5$ cells were seeded into four 250 mm dishes in each group. After 3 h incubation to allow attachment of cells, various drugs were added; control cultures were treated with diluents. The pH of the medium was checked periodically. After varying periods of exposure to drugs, cells in one dish from each group were trypsinised, resuspended and counted. Serial dilutions of cells were then plated in triplicate in α-MEM + 10% FBS. After 8–9 days, colonies were stained with methylene blue and counted. The surviving fraction (SF) was calculated according to:

$$SF = \frac{\text{cell number in suspension } \textit{treated}}{\text{cell number in suspension } \textit{control}} \times \frac{\text{plating efficacy } \textit{treated}}{\text{plating efficacy } \textit{control}}$$

Here the control conditions refer to untreated cells at $pH_e$ 7.2. To evaluate the influence of small differences in $pH_e$ on cell survival, some experiments were also performed at $pH_e$ 6.7 and 6.9.

### *Measurement of the activity of $Na^+/H^+$ and $Na^+$-dependent $HCO_3^-/CL^-$ exchangers*

Intracellular pH ($pH_i$) was measured as described previously (Boyer and Tannock, 1992). Briefly, cells grown as a monolayer on glass coverslips were loaded with BCECF-AM. The coverslip was then placed into a cuvette using a specially designed holder aligned at an angle of 30° to the excitation beam. Fluorescence was determined using an Aminco Bowman Series 2 luminescence spectrometer (SLM Instrument, NY, USA) with excitation and emission wavelengths set to 495 nm and 525 nm respectively. To determine the activities of the $Na^+/H^+$ antiport and the $Na^+$-dependent $HCO_3^-/Cl^-$ exchanger, cells were first acidified to $pH_i$ approximately 6.5 or 6.8 by placing cells in NMG containing ammonium chloride for 30 min followed by replacement with NMG. The activity of the $Na^+/H^+$ antiport in the presence or absence of EIPA was quantified by adding sodium chloride to the cuvette. Activity of the $Na^+$-dependent $HCO_3^-/Cl^-$ exchanger in the presence or absence of DIDS was quantified by adding sodium bicarbonate to the cuvette in the presence of EIPA. $H^+$ efflux via each exchanger was calculated as described previously (Boyer and Tannock, 1992).

### *Long-term exposure to drugs* in vivo

KHT or EMT-6 tumours were generated by intramuscular injection of $1 \times 10^6$ cells into the left hind leg of syngeneic C3H/HeJ or Balb/c mice respectively. Growth of tumour was monitored by passing the tumour-bearing leg through a strip of lucite with graded size holes, and tumour weight was estimated from the diameter of the tumour-bearing leg using a previously defined calibration curve. Mice were used in experiments when their tumours had grown to about 0.5 g in weight, which took approximately 7 days.

Alzet micro osmotic pumps (model 1003D, Alza, CA, USA), with a capacity of 100 μl and which pumped the fluid at a steady rate of 1 μl h$^{-1}$ for 72 h, were implanted into mice. The pumps were loaded with various concentrations of nigericin with or without hydralazine. In some experiments, DIDS and/or EIPA were placed in a separate pump since nigericin + hydralazine and DIDS formed an insoluble precipitate if combined together at high concentration. The pumps containing nigericin were transplanted into the abdominal cavity of tumour-bearing mice and those containing DIDS were implanted into the subcutaneous region of the dorsum of the mice. Tumour size and body weight were measured daily.

Four days after implantation of the pump(s), i.e. 24 h after termination of the period of continuous infusion of drugs, each tumour was excised, weighed, and minced with scissors in phosphate-buffered saline (PBS). A single-cell suspension was obtained by enzymatic digestion with trypsin (Difco) and DNAase I (Sigma) and dye-excluding cells were counted with a haemocytometer. The suspensions were diluted and plated in triplicate in α-MEM + 10% FBS (Hasuda *et al.*, 1994). After 8–10 days, cells were fixed and stained with methylene blue and colonies were counted. Surviving fraction per tumour was calculated according to:

$$SF/\text{tumour} = \frac{(\text{cells/gram } \textit{treated}\ ) \times \text{ tumour weight } \textit{treated}}{\text{cells/gram } \textit{control}\ ) \times \text{ tumour weight } \textit{control}} \times \frac{\text{plating efficacy } \textit{treated}}{\text{plating efficacy } \textit{control}}$$

### *Measurement of tissue pHe*

Mice bearing the KHT or EMT-6 tumours were anaesthetised with sodium pentobarbital given i.p. at a dose of 50 mg kg$^{-1}$. The $pH_e$ was measured using a miniature glass electrode in a 21 gauge needle (model MI-408B, Microelectrodes) against a silver–silver chloride reference electrode (model MI-402, Microelectrodes) using a pH-meter (model pH103, Corning). The reference electrode was inserted subcutaneously on the back and the pH electrode was inserted in the tumour or muscle after incising the overlying skin. Measurements of $pH_e$ were made at 50 to 75 μm increments along a single track at a depth of 3–5 mm into the tumour. A minimum of three $pH_e$ measurements per tumour were recorded (Newell *et al.*, 1993). The effect of continuous infusion of hydralazine on tumour $pH_e$ was evaluated in comparison with bolus injection. The dose of hydralazine by bolus peritoneal injection was 10 mg $kg^{-1}$. For continuous infusion, the concentration of hydralazine in osmotic pumps that were implanted in the peritoneal cavity was 12.5 mg $ml^{-1}$.

### Results

### *Assessment of exchangers*

Table I shows rates of $H^+$ efflux in KHT and EMT-6 cells. When the cells were acidified to $pH_i$ 6.5, the rate of $H^+$ efflux via the $Na^+/H^+$ exchanger was higher than that via the $Na^+$-dependent $HCO_3^-/Cl^-$ exchanger in both cell lines. As expected (Boyer and Tannock, 1992), the activity of both exchangers tended to be lower in the cell lines at $pH_i$ 6.8 than at $pH_i$ 6.5, but the $Na^+$-dependent $HCO_3^-/Cl^-$ exchanger was then quantitatively more important.

### In vitro *studies*

During 72 h exposure, the $pH_e$ of medium was relatively stable with maximum changes of < 0.1 pH units from baseline at $pH_e$ 7.2 and $pH_e$ 6.8. Figure 1a shows cell growth during long-term exposure to nigericin (0.033 $\mu$M) with or without EIPA (0.5 $\mu$M), these doses are about one-tenth of the minimum doses that are cytotoxic following short-term (i.e. up to 6 h) exposure at $pH_e$ 6.5. Minimal suppression of cell growth was observed with drug treatment at $pH_e$ 7.2. Control cells at $pH_e$ 6.8 grew slowly, and there was loss of cells exposed to nigericin ± EIPA at $pH_e$ 6.8. Surviving fractions under these conditions are shown in Figure 1b. Exposure of controls for 48–72 h to $pH_e$ 6.8 led to a surviving fraction of 0.03–0.10 in multiple experiments. Nigericin (0.033 $\mu$M) caused time-dependent cell killing of KHT tumour cells at $pH_e$ 6.8 with a survival fraction of 0.0017 at 72 h as compared with untreated cells at $pH_e$ 7.2 (surviving fraction approximately 0.06 as compared with untreated cells at $pH_e$ 6.8). Cell killing was increased minimally in the presence of EIPA.

The dose-dependent effects of EIPA and DIDS combined with nigericin (0.033 $\mu$M) on the survival of KHT cells are shown in Figure 2: Only minor effects were observed for EIPA at concentrations up to 5 $\mu$M (Figure 2a). A concentration of 0.4 mM DIDS was highly toxic to cells when used alone, and 0.1 mM DIDS enhanced cytotoxicity of nigericin with survival fraction reduced approximately 100-fold compared with nigericin alone (Figure 2b).

The dose-dependent effects of nigericin on surviving fraction of KHT and EMT-6 cells following 72 h exposure at $pH_e$ 6.8 or 7.2 are shown in Figure 3a and 3b respectively. Cell killing by nigericin was enhanced in the presence of 0.05 mM DIDS, and by DIDS + EIPA (5 $\mu$M).

The effects of $pH_e$ on survival of KHT and EMT-6 cells following 72 h exposure to nigericin with or without DIDS and EIPA are shown in Figure 4. Cell killing is very sensitive

**Figure 1** Growth (**a**) and survival (**b**) of KHT cells during 72 h exposure to nigericin 0.033 $\mu$M with or without EIPA 0.5 $\mu$M at $pH_e$ 7.2 or 6.8 *in vitro*. Open symbols are for data at $pH_e$ 7.2 and closed symbols are for data at $pH_e$ 6.8. □, Control (diluent only); ◇, nigericin alone; ○, nigericin and EIPA. Each point represents the mean ± standard deviation from three experiments.

**Table I** Rate of $H^+$ efflux (in mM $H^+$ $min^{-1}$) in KHT and EMT-6 cells

| *Activity of exchangers* | *$pH_i$ after acidification* $pH_i$ *6.5* | *$pH_i$ 6.8* |
|---|---|---|
| KHT | | |
| $Na^+/H^+$ exchanger | 7.0 ± 0.2 | 1.6 ± 0.2 |
| $Na^+$-dependent $HCO_3^-/Cl^-$ exchanger | 6.3 ± 0.3 | 4.1 ± 0.4 |
| Both combined | 13.9 ± 0.6 | 6.9 ± 0.6 |
| EMT-6 | | |
| $Na^+/H^+$ exchanger | 5.9 ± 0.2 | 1.8 ± 0.2 |
| $Na^+$-dependent $HCO_3^-/Cl^-$ exchanger | 3.7 ± 0.3 | 4.0 ± 0.4 |
| Both combined | 10.3 ± 0.5 | 8.0 ± 0.7 |

Results have been corrected for differences in buffering capacity.

**Figure 2** Effects of varying concentration of EIPA (**a**) or DIDS (**b**) alone or with nigericin (0.033 μM) on survival of KHT cells after 72 h exposure *in vitro*. Open symbols are for data at $pH_e$ 7.2 and closed symbols are for data at $pH_e$ 6.8. □, Without nigericin; ○, with nigericin. Points represent means ± standard deviation from three or more experiments.

**Figure 3** Effect of varying concentration of nigericin on survival of KHT (**a**) and EMT-6 (**b**) cells after 72 h exposure with or without DIDS (50 μM) or DIDS plus EIPA (5 μM). Open symbols are for data at $pH_e$ 7.2 and closed symbols are for data at $pH_e$ 6.8. □, Nicericin alone; ◇, nigericin with DIDS; ○, nigericin with DIDS and EIPA. Points represent means ± standard deviation from three experiments.

to $pH_e$, with a decrease in survival of 10-fold or greater for a decrement of 0.1 pH unit. At $pH_e$ 6.7, survival of KHT cells exposed to nigericin + DIDS with or without EIPA was below the limit of detection.

### In vivo *experiments*

Micro-osmotic pumps were loaded with 0.6, 2.0 or 6.0 mg $ml^{-1}$ nigericin and implanted into mice. The estimated total release of nigericin over 72 h is about 2.2, 7.2 and 22 mg $kg^{-1}$ body weight, and all of these doses were tolerated by mice. No severe early weight loss or late side-effects up to 2 weeks were observed. The maximum tolerated dose is close to 22 mg $kg^{-1}$, as when DIDS (10 mg $ml^{-1}$) was added to the pumps death of the animals was observed; this compared with a maximum tolerated dose of 2.5 mg $kg^{-1}$ nigericin by bolus injection (Hasuda *et al.*, 1994). Neither weight loss nor abnormal behaviour was observed with administration up to 2 mg $ml^{-1}$ nigericin and 10 mg $ml^{-1}$ DIDS from the osmotic pumps.

Our unpublished data on pharmacokinetics of EIPA show a half-life of about 30 min in plasma of mice with slow conversion to the less potent amiloride, the concentration of which is still lower than that of EIPA up to 2 h later. Although EIPA administered from osmotic pumps containing a concentration of 3 mg $ml^{-1}$ was not toxic to mice, the combination of nigericin + DIDS + hydralazine + EIPA caused death of Balb/c mice implanted with EMT-6 tumours.

The growth of KHT tumours during continuous 72 h exposure to various concentrations of nigericin with or without DIDS is shown in Figure 5a. Continuous administration of nigericin caused significant delay of tumour growth, which was enhanced in the presence of DIDS. The mean rate of regrowth was also slower than that of control tumours after these treatments. Figure 5b shows the surviving fraction per tumour: nigericin caused dose-dependent killing of KHT cells in mice when given by 72 h infusion, although the effect of nigericin at the highest tolerated dose was to reduce survival only to approximately $10^{-1}$. There was slight enhancement of cell killing in the presence of DIDS. The relationship between growth delay and surviving fraction is known to be compex owing to cell killing, environmental effects on potentially lethal damage, anti-proliferative effects and the proliferation and removal of damaged cells. The differences in survival fraction between each treated group and control shown in Figure 5b are consistent with or greater than the difference in tumour weight after 72 h treatment, which is shown in Figure 5a.

**Figure 4** Effect of $pH_e$ on survival of KHT (**a**) or EMT-6 (**b**) cells exposed for 72 h to various agents. □, control (diluent only); ◇, nigericin (0.033 μM); ○, nigericin with DIDS (50 μM); △, nigericin with DIDS and EIPA (5 μM). Each point represents mean ± standard deviation from three experiments.

*Measurement of tumour $pH_e$ and acidification by hydralazine*

Measurements of $pH_e$ in KHT tumours are indicated in Figure 6. Estimates of $pH_e$ after administration of hydralazine were significantly lower than estimates of $pH_e$ without treatment ($P<0.05$, paired $t$ test). The fall in tumour

**Figure 5** Growth of KHT tumours in mice during continuous infusion of agents from osmotic pumps. □, Diluents alone in pump; ◇, 0.6 mg ml$^{-1}$ nigericin; ○, 2.0 mg ml$^{-1}$ nigericin; △, 6.0 mg ml$^{-1}$ nigericin. Open and closed symbols represent nigericin alone and nigericin with 10 mg ml$^{-1}$ DIDS respectively. Each point represents mean ± standard deviation from four or more tumours. (**b**) Surviving fraction per KHT tumours after 72 h continuous infusion of varying concentration of nigericin with or without 10 mg ml$^{-1}$ DIDS using osmotic pumps. □, Nigericin alone; ●, nigericin and DIDS. Each point represents mean ± standard deviation from three or more tumours.

$pH_e$ after bolus injection was observed only up to 4 h with a maximal decrease at 2 h after injection, whereas that obtained by continuous infusion lasted throughout the period of infusion.

Table II indicates surviving fractions of cells in KHT and EMT-6 tumours of mice treated with continuous 72 h infusions of nigericin, hydralazine and DIDS via two infusion pumps. Mice were able to tolerate this combination of drugs and there were no animal deaths up to 2 weeks. The combination of nigericin and hydralazine caused an approximately 2-fold increase in cell killing as compared with nigericin alone. If DIDS was given with this combination of agents, the surviving fraction per tumour was reduced to about 0.02–0.03.

When EIPA was added to the second osmotic pump together with DIDS, this led to death of Balb/c mice implanted with EMT-6 tumours. C3H mice bearing KHT tumours were able to tolerate this treatment. EIPA caused a marked augmentation in cell killing by the combination of nigericin, DIDS and hydralazine with a surviving fraction per tumour reduced approximately 10-fold compared with the combination of the drugs without EIPA.

### Discussion

The ionophore nigericin, which lowers $pH_i$ by allowing exchange of intracellular $K^+$ for extracellular $H^+$, has been shown to be cytotoxic to cultured cells at $pH_e \leqslant 6.5$ (Rotin *et al.*, 1987). The average values of $pH_e$ in solid tumours, however, are usually about $pH_e$ 6.9, which is only 0.4–0.5 pH units lower than those in normal tissues (Wike-Hooley *et al.*, 1984). These values can be lowered slightly by vasodilator drugs (Newell *et al.*, 1992) or by infusion of glucose with or without insulin (Hwang *et al.*, 1991; Jähde *et al.*, 1992). In the present study, $pH_e$ 6.8 was selected as representative of the $pH_e$ that might be achieved in tumours, and long-term exposures of up to 72 h to low concentrations of nigericin were shown to kill tumour cells at $pH_e$ 6.8 *in vitro*. This observation indicates the potential for continuous administration of nigericin to kill cells in solid tumours as compared with normal tissues based on differences in $pH_e$ (6.8 *vs* 7.2).

We attempted to enhance the cytotoxicity of nigericin with DIDS, which is an inhibitor of the $Na^+$-dependent $HCO_3^-$/$Cl^-$ exchanger, and with EIPA, which is an inhibitor of the $Na^+/H^+$ exchanger, as both of the exchangers may have important roles in regulating $pH_i$ under acidic conditions (Cassel *et al.*, 1988; Grinstein *et al.*, 1989). In previous experiments, short-term exposure to EIPA or DIDS increased the killing of tumour cells by nigericin (Maidorn *et al.*, 1993; Luo and Tannock, 1994) when used at $pH_e$ 6.5. We measured the activity of both exchangers and confirmed our previous

**Figure 6** Extracellular pH ($pH_e$) in KHT tumours with or without treatment with hydralazine by bolus injection (◆) or by continuous infusion (○). □, Control. The dose of hydralazine by bolus injection was 10 mg kg$^{-1}$. The concentration of hydralazine in osmotic pumps was 12.5 mg ml$^{-1}$, leading to delivery of approximately 36 mg kg$^{-1}$ body weight over 72h (i.e. 12.5 μg h$^{-1}$) Values are mean ± s.d. Each point comprised four or more tumours. Values at 2 h after bolus injection, and at 24 h and 48 h after start of the infusion, are significantly different from control ($P<0.05$, paired *t*-test).

results, which indicate that the activity of the $Na^+$-dependent $HCO_3^-/Cl^-$ exchanger is quantitatively more important in regulating $pH_i$ at values of $pH_e$ just below the physiological range (Boyer and Tannock, 1992). Consistent with this observation, we found that EIPA alone enhanced cell killing of KHT cells by nigericin only slightly at $pH_e$ 6.8, whereas DIDS caused a greater enhancement of cell killing by nigericin. However, if EIPA was given in combination with nigericin and DIDS, there was increased cell killing at $pH_e$ 6.8 in culture, so there may be therapeutic potential from pharmacological inhibition of both exchangers.

We demonstrated that small differences in $pH_e$ between pH 6.7 and 6.9 greatly affected the degree of killing by nigericin with or without EIPA or DIDS *in vitro*. This exquisite sensitivity to $pH_e$ suggests that there is considerable potential in combining the current approaches with treatments that lower $pH_e$ in tumour tissue.

Our *in vivo* experiments have shown that chronic administration of nigericin can lead to a decrease in surviving fraction in both KHT and EMT-6 tumours, and that this effect is augmented by hydralazine, which inhibits tumour blood flow and lowers tumour $pH_i$ as well as $pH_e$ (Bhujwalla *et al.*, 1990). The increase in cell killing caused by hydralazine could be due to acidification of tumour or to

**Table II** Surviving fraction per tumour for KHT and EMT-6 tumours following various treatments

| *Treatment* | *Tumour weight (g)* | *No. of cells g$^{-1}$ (× 10$^7$)* | *SF/tumour* |
|---|---|---|---|
| KHT | | | |
| Contol | 1.17±0.45 | 1.68±0.52 | 1.0 |
| Nigericin 2.0 mg ml$^{-1}$ | 0.75±0.10 | 1.21±0.37 | 0.18±0.07 |
| Hydralazine 12.5mg ml$^{-1}$ | 0.85±0.12 | 1.23±0.44 | 0.40±0.08 |
| Nigericin 2.0 mg ml$^{-1}$ + hydralazine 12.5 mg ml$^{-1}$ | 0.55±0.18 | 0.79±0.26 | 0.10±0.06 |
| Nigericin 2.0 mg ml$^{-1}$ + hydralazine 12.5 mg ml$^{-1}$ + DIDS 10 mg ml$^{-1}$ | 0.52±0.06 | 0.45±0.07 | 0.03±0.01 |
| Nigericin 2.0 mg ml$^{-1}$ + hydralazine 12.5 mg ml$^{-1}$ + DIDS 10 mg ml$^{-1}$ + EIPA 3 mg ml$^{-1}$ | 0.30±0.11 | 0.05±0.02 | 0.002±0.001 |
| EMT-6 | | | |
| Control | 1.12±0.06 | 1.48±0.49 | 1.0 |
| Nigericin 2.0 mg ml$^{-1}$ + hydralazine 12.5 mg ml$^{-1}$ | 0.95±0.05 | 0.71±0.24 | 0.22±0.08 |
| Nigericin 2.0 mg ml$^{-1}$ + hydralazine 12.5 mg ml$^{-1}$ + DIDS 10 mg ml$^{-1}$ | 0.57±0.11 | 0.18±0.11 | 0.02±0.01 |

The drugs were given by 72 h infusion from micro-osmotic pumps and the concentration of each drug in the pump is indicated. Values are mean ± s.d. Each group comprised four or more tumours from three or more individual experiments.

trapping of the drugs within the tumour as a result of decreased blood flow (Parkins *et al.*, 1994).

We found that DIDS enhanced cell killing *in vitro* by nigericin, although cell survival was reduced only to approximately 0.02 at tolerated doses in EMT-6 tumours in the presence of hydralazine. The effect of DIDS was greater than that in the absence of hydralazine. This observation is consistent with results obtained *in vitro*, which showed that DIDS enhanced cell killing by nigericin at lower $pH_e$. Although we could apply EIPA only to C3H/HeJ mice by continuous infusion, the greatest cell killing was observed when both exchangers were inhibited, consistent with results obtained in culture. This suggests the importance of inhibition of both the $Na^+/H^+$ antiport and the $Na^+$-dependent $HCO_3^-/Cl^-$ exchanger in maximising this approach to tumour therapy.

There is evidence that the acute administration of agents that acidify cells may enhance the effects of hyperthermia against experimental tumours (Miyakoshi *et al.*, 1986; Ruifrok *et al.*, 1987; Kim *et al.*, 1991; Song *et al.*, 1993). Our current results using chronic administration of such agents might have relevance to studies of hyperthermia, although repeated or prolonged heat treatments within 72 h would only be useful if thermotolerance were inhibited.

The potential for using continuous infusion of drugs in patients is greater than in small animals, in which multiple infusions are technically difficult. Our experiments have shown; (i) the feasibility of selective cell killing in culture at $pH_e$ 6.8, a value that may be representative for some regions of solid tumours, and (ii) the ability to obtain anti-tumour effects in an animal model. The exquisite sensitivity of cell survival to $pH_e$ at values close to 6.8 suggests the potential for enhancing therapeutic effects through mechanisms that lower tumour pHe slightly and/or for using alternative measures to kill non-acidic tumour cells. Such experiments are in progress in our laboratory.

**Abbreviations**

EIPA, 5-(*N*-ethyl-*N*-isopropyl) amiloride; DIDS, 4,4-diisothiocyanstilbene 2,2-disulphonic acid; αMEM alpha-minimum essential medium; BCECF-AM 2′,7′-bis-(2-carboxyethyl)-5-(and 6)-carboxyfluorescein acetoxymethyl ester; FBS, fetal bovine serum; $pH_e$, extracellular pH; $pH_i$, intracellular pH.

**Acknowledgements**

We thank Dr Pedro Salinas for his helpful advice and Ms Carol Lee for technical assistance. This work is supported by a research grant from the Medical Research Council of Canada.

### References

ARONSON PS, NEE J AND SUHM MA. (1982). Modifier role of internal $H^+$ in activating the $Na^+-H^+$ exchanger in renal microvillus membrane vesicles. *Nature*, **299**, 161–163.

BHUJWALLA ZM, TOZER GM, FIELD SB, MAXWELL RJ AND GRIFFITHS JR. (1990). The energy metabolism of RIF-1 tumours following hydralazine. *Radiother. Oncol.*, **19**, 281–291.

BOYER MJ AND TANNOCK IF. (1992). Regulation of intracellular pH in tumour cell lines: influence of microenvironmental conditions. *Cancer Res.*, **52**, 4441–4447.

CASSEL D, SCHARF O, ROTMAN M, CRAGOE JR EJ AND KATZ M. (1988). Characterization of $Na^+$-linked and $Na^+$-independent $Cl^-/HCO_3^-$ exchange systems in Chinese hamster lung fibroblastoma. *J. Biol. Chem.*, **263**, 6122–6127.

GILLIES RJ, LIU Z AND BHUJWALLA Z. (1994). $^{31}P$-MRS measurements of extracelluar pH of tumors using 3-aminopropylphosphonate. *Am. J. Physiol.*, **267**, C195–203.

GRINSTEIN S, ROTIN D AND MASON MJ. (1989). $Na^+/H^+$ exchanger and growth factor-induced cytosolic pH changes. Role in cellular proliferation. *Biochim Biophys. Acta*, **988**, 73–97.

HASUDA K, LEE C AND TANNOCK IF. (1994). Anti-tumor activity of nigericin and 5-(*N*-ethyl-*N*-isopropyl) amiloride: an approach to therapy based on cellular acidification and the inhibition of regulation of intracellular pH. *Oncol. Res.*, **6**, 259–268.

HWANG YC, KIM S-G, EVELHOCK JL, SEYEDSADR M AND ACKERMANN JH. (1991). Modulation of murine radiation-induced fibrosarcoma-1 tumor metabolism and blood flow *in situ* via glucose and mannitol administration monitored by $^{31}P$ and $^{2}H$ nuclear magnetic resonance spectroscopy. *Cancer Res.*, **51**, 3108–3118.

JÄHDE E, VOLK T, ATEMA A, SMETS LA, GLÜSENKAMP K-H AND RAJEWSKY MF. (1992). pH in human tumor xenografts and transplanted rat tumors: effect of insulin, inorganic phosphate, and m-iodobenzylguanidine. *Cancer Res.*, **52**, 6209–6215.

JOHNSON JD AND EPEL D. (1976). Intracellular pH and activation of sea urchin eggs after fertilizaton. *Nature*, **304**, 371–374.

KIM GE, LYONS JC AND SONG CW. (1991). Effects of amiloride on intracellular pH and thermosensitivity. *Int. J. Radiat. Oncol. Biol. Phys.*, **20**, 541–549.

L'ALLEMAIN G, PARIS S AND POUYSSÉGUR J. (1985). Role of a $Na^+$-dependent $Cl^-/HCO_3^-$ exchanger in regulation of intracellular pH in fibroblasts. *J. Biol. Chem.*, **260**, 4877–4883.

LUO J AND TANNOCK IF. (1994). Inhibition of the regulation of intracellular pH: potential of 5-(*N,N*-hexamethylene) amiloride in tumour selective therapy. *Br. J. Cancer*, **70**, 617–624.

LYONS JC, ROSS BD AND SONG CW. (1993). Enhancement of hyperthermia effect in vivo by amiloride and DIDS. *Int. J. Radiat. Oncol. Biol. Phys.*, **25**, 95–103.

MAIDORN RP, CRAGOE JR EJ AND TANNOCK IF. (1993). Therapeutic potential of analogues of amiloride: inhibition of the regulation of intracellular pH as a possible mechanism of tumour selective therapy. *Br. J. Cancer*, **67**, 297–303.

MIYAKOSHI J, ODA W, HIRATA M, FUKUHORI N AND INAGAKI C. (1986). Effects of amiloride on thermosensitivity of Chinese hamster cells under neutral and acidic pH. *Cancer Res.*, **46**, 1840–1843.

MOOLENAAR WH, TERTOOLEN LGJ AND DE LAAT SW. (1984). The regulation of cytoplasmic pH in human fibroblasts. *J. Biol. Chem.*, **259**, 7563–7569.

NEWELL K, WOOD P, STRATFORD I AND TANNOCK I. (1992). Effects of agents which inhibit the regulation of intracellular pH on murine solid tumours. *Br. J. Cancer*, **66**, 311–317.

NEWELL K, FRANCHI A, POUYSSÉGUR J AND TANNOCK I. (1993). Studies with glycolysis-deficient cells suggest that production of lactic acid is not the only cause of tumor acidity. *Proc. Natl Acad. Sci. USA*, **90**, 1127–1131.

PARKINS C, CHADWICK JA AND CHAPLIN DJ. (1994). Enhancement of chlorambucil cytotoxity by combination with flavone acetic acid in a murine tumour. *Anticancer Res.*, **14**, 1603–1608.

RUIFROK ACC AND KONINGS AWT. (1987). Effects of amiloride on hyperthermic cell killing of normal and thermotolerant mouse fibroblast LM cells. *Int. J. Radiat. Biol.*, **52**, 385–392.

ROTIN D, WAN P, GRINSTEIN S AND TANNOCK I. (1987). Cytotoxity of compounds that interfere with the regulation of intracellular pH: a potential new class of anticancer drugs. *Cancer Res.*, **47**, 1497–1504.

ROTIN D, STEELE-NORWOOD D, GRINSTEIN S AND TANNOCK I. (1989). Requirement of the $Na^+/H^+$ exchanger for tumor growth. *Cancer Res.*, **49**, 205–210.

SONG WC, LYONS JC, GRIFFIN RJ, MAKEPEACE CM AND CRAGOE JR EJ. (1993). Increase in thermosensitivity of tumor cells by lowering intracellular pH. *Cancer Res.*, **53**, 1599–1601.

SPARKS RL, POOL TB, SMIKTH NKR AND CANERON IL. (1983). Effects of amiloride on tumor growth and intracellular element contact of tumor cells *in vitro*. *Cancer Res.* **43**, 73–77.

SZOLGAY-DANIEL E, CARLSSON J, ZIEROLD K, HOLTERMANN G, DUFAU E AND ACKER H. (1991). Effects of amiloride treatment on U-118 MG and U-251 MG human glioma and HT-29 human colon carcinoma cells. *Cancer Res.*, **51**, 1039–1044.

TANNOCK IF. (1968). The relation between cell proliferation and vascular system in a transplanted mouse mammary tumour. *Br. J. Cancer*, **22**, 258–273.

TANNOCK IF AND ROTIN D. (1989). Acid pH in tumors and its potential for therapeutic exploitation. *Cancer Res.*, **49**, 4373–4384.

THOMAS RC. (1977). The role of bicarbonate, chloride and sodium ions in the regulation of intracellular pH in snail neurones. *J. Physiol.*, **273**, 317–338.

VAUPEL P, KALLINOWSKY F AND OKUNIEFF P. (1989). Blood flow, oxygen and nutrient supply, and metabolic microenvironment of human tumors: a review. *Cancer Res.*, **49**, 6449–6465.

WIKE-HOOLEY JL, HAVEMAN J AND REINHOLD JS. (1984). The relevance of tumour pH to the treatment of malignant disease. *Radiother. Oncol.*, **2**, 343–366.